# 1234. Can Susceptibility to One Carbapenem be Conferred to Another? Frequency of Discordance in Gram-negative Clinical Isolates

**DOI:** 10.1093/ofid/ofab466.1426

**Published:** 2021-12-04

**Authors:** Pam M Ku, Diana A Hobbs, Melissa Gilmore, Athena L Hobbs

**Affiliations:** 1 Augusta University Medical Center, Augusta, Georgia; 2 Washington University School of Medicine, St. Louis, Missouri; 3 Baptist Memorial Hospital, Columbus, Mississippi; 4 Methodist University Hospital, Memphis, Tennessee

## Abstract

**Background:**

Carbapenem-Resistant Enterobacterales (CRE) and Carbapenem-Resistant *Pseudomonas aeruginosa* (CRPA) can exhibit resistance to one carbapenem while remaining susceptible to another. While case reports describing discrepant carbapenem susceptibilities are available, the authors are unaware of any literature reporting aggregate carbapenem susceptibility discrepancies at a hospital level.

**Methods:**

Susceptibility data from April 1, 2017 - December 31, 2017 was extracted through an antibiogram report for a 706-bed hospital. Ertapenem, imipenem-cilastatin, and meropenem susceptibilities were captured and compared for common Enterobacterales and *Pseudomonas aeruginosa.* Organism identification was performed using Matrix Assisted Laser Desorption Ionization-Time of Flight (MALDI-TOF) mass spectrometry. Antibiotic susceptibility testing was performed using BD Phoenix^TM^. Carbapenem susceptibilities were interpreted using the most updated Clinical and Laboratory Standards Institute (CLSI) breakpoints at the time of assessment (2021). Carbapenem discordance was defined as an organism being susceptible to one carbapenem and non- susceptible (intermediate or resistant) to another. Approval was obtained from the institution’s Institutional Review Board.

**Results:**

Meropenem proved to be the most active antimicrobial for all organisms (Figure 1). Carbapenem susceptibility discordance ranged from 0%-23.8% (Table 1). There was a significant difference in the incidence of discordance between Enterobacterales and *Pseudomonas aeruginosa* isolates (2.6% vs. 6.1%, p < 0.001). Of the 20 *Pseudomonas aeruginosa* isolates with discordant carbapenem susceptibilities, 70% were meropenem susceptible/imipenem non-susceptible and 30% were imipenem susceptible/meropenem non-susceptible. The most common site for discordance was urine for both Enterobacterales and *Pseudomonas aeruginosa*. However, while there was a significant rate of discordance between sites for *Pseudomonas* isolates, this was not the case for Enterobacterales (Table 2).

Figure 1: Carbapenem Susceptibility by Isolate

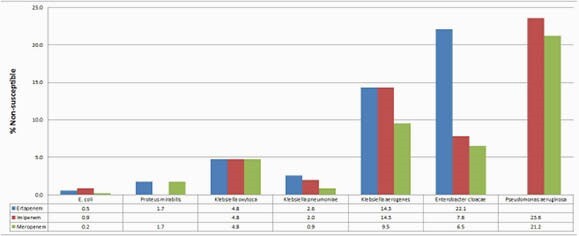

Table 1: Frequency of Carbapenem Discordance

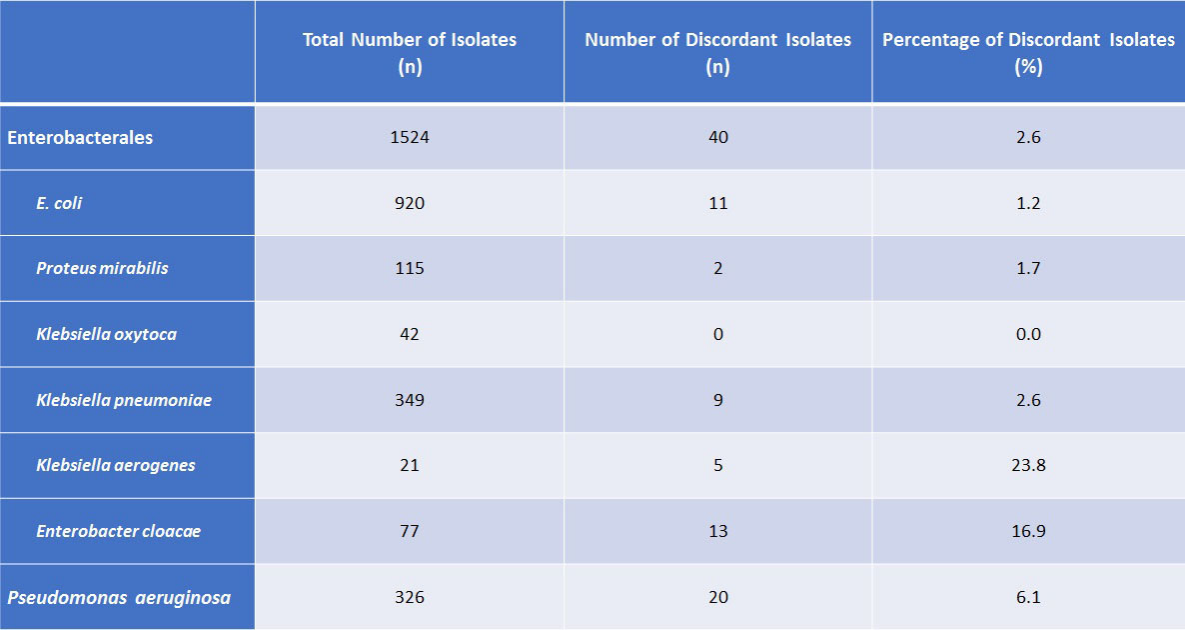

Table 2: Frequency of Carbapenem Discordance by Site

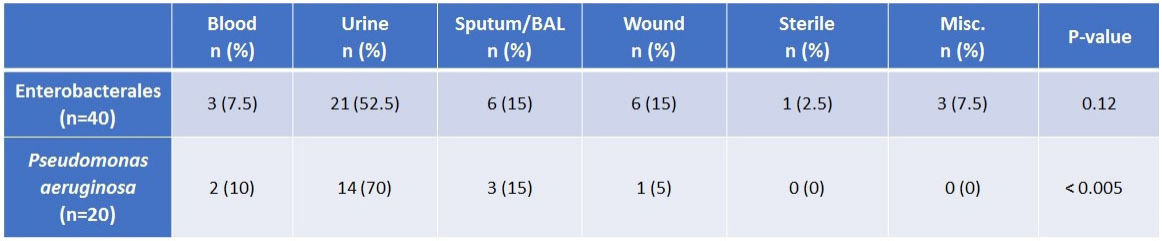

**Conclusion:**

Due to the wide range of susceptibility discordance, clinical implications can be drastic if an institution is relying on susceptibility of one carbapenem to confer susceptibility to another carbapenem.

**Disclosures:**

**All Authors**: No reported disclosures

